# The Hospitals Association

**Published:** 1886-11-20

**Authors:** 


### The Hospitals Association: The Conversazione.

Introductory.

The *conversazione* of the members and friends of the Hospitals Association, held on Wednesday last at the Rooms of the Medical Society of London, marks, we believe, an epoch of no small magnitude in the career of this Institution. The distinguished and representative gathering which was brought together at once testified the fact that men of all walks and professions of life are beginning to take a practical interest in our charities. The *conversazione* was, so to speak, a living testimony to the value of the work which the Hospitals Association has undertaken to do in the interest of the community at large, and it showed that the institution, young as it is, has not only its *raison d'être*, but has won the approval of the public.

Origin of the Hospitals Association.

Before we proceed to describe the interesting proceedings of Wednesday, we may, perhaps, not be deemed discursive if we refer for a moment to the origin of the Association itself. In the summer of 1883 a Hospital Conference was held, which resulted in the appointment of a Committee of Hospital Managers to consider what steps should be taken to secure some method of combined action among hospitals. This Committee worked hard for some time, and after great deliberation they arrived at the conclusion that an Association of Hospital and Asylum Managers was highly desirable and necessary.

Meeting at the Mansion House.

As a result of this Committee's work, before the year 1883 was numbered with the past, the constitution of the Hospitals Association was prepared and adopted. On 1st February 1884 a meeting of a very influential character was held at the Mansion House, the chair being taken by the Lord Mayor, Mr. Alderman Fowler, M.P. At this inaugural meeting nearly everyone of "light and leading" in the hospital world was present. The cogent reasoning of some of the speakers made it very clear indeed that there was "ample room and verge enough" for the institution of an Association of the kind indicated. Sir T. Fowell Buxton, in moving the resolution in support of the establishment of the Hospitals Association, dwelt with peculiar force on some of the points most sorely needing reform. Everyone left the meeting fully convinced that the Hospitals Association was a want which circumstances had rendered a necessity, and that there lay before it a field of operation almost illimitable in extent.

The "Child Born."

The infant born at the Mansion House on 1st February 1884 proved a remarkably healthy child. There were not wanting, even at that time, prognosticators who foretold the advent either of an abortion, or at best of a sickly infant, which would perish from sheer inanition. Job's comforters are to be found in every century and in every clime, and even to-day there are some who try to raise the cry that the Hospitals Association is dead. If such persons could have looked in upon the brilliant gathering on Wednesday, they would probably now be prepared to admit that the child is of a more vigorous growth than they anticipated.

Its Purposes.

The Hospitals Association has before it a clearly defined object. It seeks to be a body whose members shall represent all the great hospitals of the country, and, through them, so to facilitate and organise hospital management that the greatest good shall be meted out to the suffering poor at the minimum of cost. The Duke of Cambridge, in an article last week in this Journal, pointed out the advantage which it is to the country that our charitable institutions are not under State control in the same way as our Poor-law institutions. If our charities were placed upon the rate-book, our alms-giving would become an ugly mulct, and Christian philanthropy would become a dead letter. The Hospitals Association saw, in the recession of public charity, that the whole subject of hospital administration required to be brought under the notice of the public, and that the attention of all classes must be aroused and fixed before adequate support would be forthcoming. A deficit of £50,000 a year is a big sum to be made up in London alone, but when the public throughout the country are satisfied by the production of facts that the expenditure of the various institutions is carefully watched over by the managers, and that resources are properly economized, this sum, we believe, will be forthcoming. The large increase in the Hospital Sunday collections this year proves this to demonstration.

**How the Matter Stands.** The question which presents itself is one of the utmost simplicity. Every hospital is a spending department, and, as such, is capable of being brought under an organised system of control or supervision. The object of every charity is—or, at any rate, ought to be—to husband its funds to the farthest extent possible. In a multitude of counsellors there is safety, and hence it follows, in a representative body such as the Hospitals Association, that facts of great practical value may be forthcoming. All institutions represented may alike benefit, and the truth of the principle, "union strong and great," will be brought home with especial force. All hospital managers are in one sense the custodians of a public purse. So long as they retain the confidence of the public, we do not doubt that the flow of charity will suffice for the needs of the necessitous, and the best way for its sure retention is, we venture to think, through an organised and recognised body of representatives.

**Unity of Action.** Perfection is a thing rarely attained in this world. In all hospitals there are defects in management, and these, we believe, may be best remedied by unity of action. Public discussion cannot fail to eliminate abuse and misdirection wherever they are found. As the Association has become more matured, the public have begun to recognise its value, and to give freely, content in the belief that the Hospitals Association forms a centre of co-operation and intercommunication between all the charities in the common interest, where the whole question of hospital and asylum administration, nursing expenditure, and management is being wisely husbanded and matured.

**The Company.** The guests were received by Sir Andrew Clark, M.D., F.R.S., President of the Hospitals Association. For over an hour there was a continuous stream of arrivals, and the spacious rooms of the Medical Society were soon so crowded that locomotion became a matter of no small difficulty. Wednesday's gathering was, as we have said, a highly representative one—representative not only of London, but of the whole country. Had the Hospitals Association attained its hundredth birthday, instead of being a juvenile institution not quite three years old, the *soirée* could hardly have been conducted with greater success. As invariably happens on such occasions, there were many absentees. Faces whom we should have liked to see were missing, but from these absent friends came a mighty pile of letters expressive of regret. The Lady Superintendents and Matrons were strongly represented, noticeable among the company being Miss Lückes, of the London; Miss Manson, of "Barts"; Miss Victoria Jones, of Guy's; Miss Rogers, of the Leicester Infirmary; Miss Wood, of the Hospital for Sick Children, Great Ormond Street; Miss Suckling, of the Royal Hants County Hospital; Miss Mackey, of the Lock Hospital, Colchester; Miss Busby, of the General Hospital, Birmingham, and many more. Among other provincial institutions represented were the Newcastle Infirmary, by Professor Philipson; the Nurses' Home and Institute, Bath, by Miss Lander; the General Hospital, Nottingham, by Mr. White; the Northampton County Asylum, by Dr. Greene; the Radcliffe Infirmary, Oxford, by Dr. Collier; the Sussex County Hospital, by Lieut.-General Bourchier; the Bootle Borough Hospital, by its Hon. Sec.; the Essex and Colchester Hospital, by Mr. Bland; the Ipswich Borough Asylum, by Mr. Winch; the Richmond Hospital, by Colonel Sparks; the Devon and Exeter Hospital, by Miss Robinson; and the Littlemore County Asylum by Major Fane. Many others connected in various ways with our great "homes of mercy" lent their support to the Association by their presence on Wednesday. Among these we noticed Sir Richard Webster, the Attorney-General, and Miss Webster; Mr. Fell-Pease, M.P.; Sir Arthur Watson; Sir Henry and Lady Knight; Lieut.-Col. Montefiore; Sir Harry Verney; and Mr. Alderman Isaacs. Among the leading members of the profession were Sir Risdon Bennett, Dr. Steele, of Guy's, Sir Joseph Fayrer, Mr. Timothy Holmes, F.R.C.S., of St. George's Hospital, Dr. Bristowe, Dr. Gilbart-Smith, and Mr. T. Moore, F.R.C.S.

**The Speeches.** Sir Andrew Clarke, in addressing the assembly, said that he gave to each and all a most cordial welcome. He welcomed with special pleasure the large and distinguished company of ladies who by their presence gave grace and brightness to the gathering. It was to him a source of the greatest possible satisfaction to see there so many ladies who were practically engaged in one way or other with the great work which the hospitals had in hand. In this work the best energies of men and women were both called into play. To it men brought the force of their robust and masculine intellect, whilst the gentler sex contributed that tenderness of sympathetic feeling which was the necessary complement of it. Without the gentleness of the womanly character, much of the great work done by hospitals would be incomplete, and, indeed, practically impossible.

Sir Sydney Waterlow dwelt upon the growing necessity for intercommunication among our hospitals with a view to the promotion, not only of their efficiency, but of the economy of their management, and he expressed his belief that the Hospitals Association would accomplish this end.

The company inspected the numerous exhibits with considerable interest. Our detailed notice of these, together with a full report of the speeches, we reserve till next week.

---

A Physician went one day to see a patient, and was told by the servant that she had just expired. "Your lady may be apparently dead," said the doctor, "yet not actually so." He alighted from his carriage, and went upstairs, where he found his patient really dead, with the customary fee in the palm of her hand, and taking it, he remarked with much seriousness, "I see the poor lady expected me. Heaven rest her soul!"

